# Efflux Inhibitor Bicalutamide Increases Oral Bioavailability of the Poorly Soluble Efflux Substrate Docetaxel in Co-Amorphous Anti-Cancer Combination Therapy

**DOI:** 10.3390/molecules24020266

**Published:** 2019-01-11

**Authors:** Adam Bohr, Thais Leite Nascimento, Necati Harmankaya, Johan Juhl Weisser, Yingya Wang, Holger Grohganz, Thomas Rades, Korbinian Löbmann

**Affiliations:** 1 Department of Pharmacy, University of Copenhagen, 2100 Copenhagen, Denmark; adam.bohr@sund.ku.dk (A.B.); thaisleite@gmail.com (T.L.N.); necatiharmankaya@gmail.com (N.H.); johan.weisser@sund.ku.dk (J.J.W.); argentwang@hotmail.com (Y.W.); holger.grohganz@sund.ku.dk (H.G.); thomas.rades@sund.ku.dk (T.R.); 2 Laboratory of Pharmaceutical Nanotechnology and Drug Delivery Systems, School of Pharmacy, Federal University of Goiás, Goiânia 74605-170, Brazil

**Keywords:** Anti-cancer, docetaxel, bicalutamide, co-amorphous, oral delivery, efflux inhibitor, in vivo

## Abstract

Many anti-cancer drugs are difficult to formulate into an oral dosage form because they are both poorly water-soluble and show poor permeability, the latter often as a result of being an intestinal efflux pump substrate. To obtain a more water-soluble formulation, one can take advantage of the higher solubility of the amorphous form of a given drug, whereas to increase permeability, one can make use of an efflux pump inhibitor. In this study, a combination of these two strategies was investigated using the co-amorphous approach, forming an amorphous mixture of two anti-cancer drugs, docetaxel (DTX) and bicalutamide (BIC). The efflux substrate, DTX, was combined with the efflux inhibitor, BIC, and prepared as a single phase co-amorphous mixture at a 1:1 molar ratio using vibrational ball milling. The co-amorphous formulation was tested in vitro and in vivo for its dissolution kinetics, supersaturation properties and pharmacokinetics in rats. The co-amorphous formulation showed a faster in vitro dissolution of both drugs compared to the control groups, but only DTX showed supersaturation (1.9 fold) compared to its equilibrium solubility. The findings for the co-amorphous formulation were in agreement with the pharmacokinetics data, showing a quicker onset in plasma concentration as well as a higher bioavailability for both DTX (15-fold) and BIC (3-fold) compared to the crystalline drugs alone. Furthermore, the co-amorphous formulation remained physically stable over 1.5 years at 4 °C under dry conditions.

## 1. Introduction

Oral administration of anti-cancer drugs is preferred over intravenous delivery as it provides a more convenient therapy for patients, who can administer the drug by themselves at home, avoiding hospitalization and reducing healthcare costs. Also, oral administration can provide increased therapeutic efficacy and reduced side effects due to more sustained and moderate drug plasma concentrations from a daily administration [1,2]. However, the oral delivery of widely used and highly effective anti-cancer drugs such as paclitaxel and docetaxel is problematic due to their low oral bioavailability [3,4]. A challenging problem with many anti-cancer drugs is that they are both poorly water-soluble and show poor permeability, the latter often because the drug is substrate to intestinal efflux pumps [5]. Thus, they are mainly administered parenterally via intravenous infusion at the clinical setting, despite the disadvantages associated with their administration. The poor solubility of these drugs is also problematic for infusions, as large volumes are required to dissolve the drug. Many of these drugs need to be dissolved in a mixture of water, ethanol and solubilizers, such as polysorbate and chremophor, the latter causing severe side effects and allergic reactions [6]. Furthermore, these drug-delivery systems need to be sterile, which is costly and carries the risk of infection, and trained staff are required for drug preparation and administration, which is reflected in high total therapy costs [7]. Thus, switching from an intravenous to an oral therapy may bring many advantages.

In order to obtain a systemic effect, both aqueous dissolution and intestinal permeability of the drug are crucial for sufficient bioavailability [8]. If the main mechanism for drug permeation is passive diffusion, increasing the solubility and free drug fraction on the intestinal side can be sufficient to increase passive permeation of the drug and hence bioavailability [9]. In order to improve drug dissolution, solubility and free drug fraction, drugs can be for example prepared in an amorphous form [10,11]. The main drawback of using highly soluble amorphous pure drugs is their inherent tendency to recrystallize into the poorly soluble crystalline form, as the amorphous form is thermodynamically unstable [12]. However, the amorphous form can be stabilized, e.g., by the formation of a co-amorphous system [11,13]. A co-amorphous system is a homogeneous amorphous single-phase mixture of two low molecular weight components. In such a system, one component is usually the drug to be stabilized in the amorphous form, whilst the second component can be an inert excipient such as an amino acid [14], but also a pharmaceutical active compound [15,16]. In the latter case, the second component may be used in combination therapy, and an amorphous stabilizer at the same time. 

Since many anti-cancer drugs are substrates to intestinal efflux pumps, however, an increased solubility may not necessarily lead to increased permeability and hence, more advanced strategies to improve their permeability are necessary. In order to inhibit undesired drug efflux and hence, increase bioavailability, one strategy is to co-administer efflux pump inhibitors, including some pharmaceutical surfactants [17,18]. This has been for example shown for the drug docetaxel, which is poorly water-soluble and an efflux pump substrate, by using the efflux-pump inhibitor Vitamin E TPGS as an excipient in a self-emulsifying drug delivery system [19] or a chemically synthesized cyclodextrin-based polymeric micelle with efflux pump inhibition properties [20].

Interestingly, while many anti-cancer drugs are substrates to the efflux pumps, some anti-cancer drugs, e.g., bicalutamide, lonafarnib and mitomycin, act as inhibitors to the efflux pump [5,21]. These efflux pumb inhibitors could thus allow increased uptake of drugs that are substrates to the efflux pumps, such as docetaxel [5]. In addition to their efflux inhibition mechanism, they may also act as inhibitors to CYP enzymes including CYP3A4 and CYP2C19, which could also increase the uptake of docetaxel [22,23,24,25]. In this study we investigate an oral anti-cancer combination therapy composed of docetaxel and bicalutamide using the co-amorphous formulation technology. The aim of the present study is to increase oral uptake and enhance the bioavailability of the poorly water-soluble and poorly permeable anti-cancer drug docetaxel [5] by combining it in a co-amorphous drug/drug system with the anti-cancer drug bicalutamide. In such a system, both drugs are intended to stabilize each other in an amorphous form and since bicalutamide is an efflux pump inhibitor, it additionally may facilitate the uptake of docetaxel in vivo. For this purpose, co-amorphous docetaxel/bicalutamide mixtures were prepared by vibrational ball milling and characterized with respect to their solid-state properties, physical stability, dissolution behavior and in vivo performance.

## 2. Results and Discussion

### 2.1. Solid State Characterization

**Figure 1:**
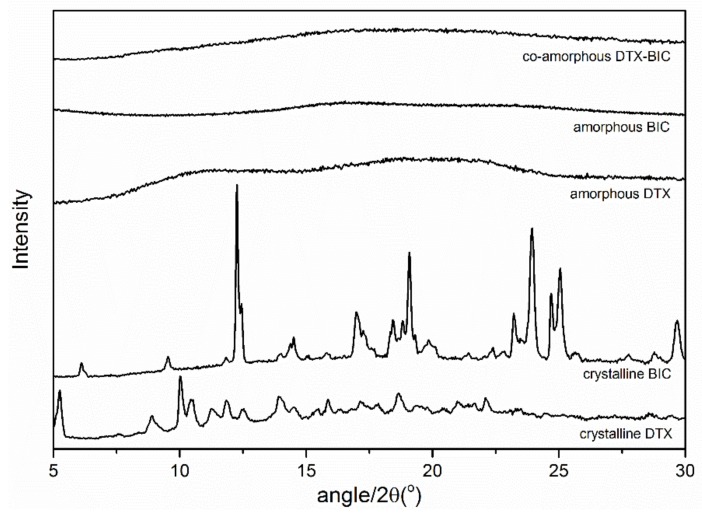
X-ray powder diffraction (XRPD) diffractograms of the bulk materials crystalline docetaxel (DTX) and crystalline bicalutamide (BIC), as well as ball-milled DTX, ball-milled BIC and the ball milled mixture of both drugs at a molar ratio 1:1.

The solid-state forms of the starting materials and the samples after ball milling were analyzed with X-ray powder diffraction (XRPD). Figure 1 shows the XRPD patterns of the crystalline drugs, the freshly prepared ball milled single drugs and the drug mixture at a 1:1 molar ratio. It can be seen that upon ball milling the characteristic diffraction peaks of the crystalline starting materials disappeared and an amorphous halo was observed for each ball milled sample, i.e. the pure drugs as well as their mixture became amorphous during the ball milling process.

Amorphization of the ball milled samples was further confirmed by the appearance of a glass transition temperature (*T_g_*) in the DSC measurements (Figure S1). The pure drugs bicalutamide (BIC) and docetaxel (DTX) had *T_g_*s at 49.8 ± 1.1 °C and 138.2 ± 2.3 °C, respectively. When ball-milled together, a single *T_g_* at 73.8 ± 0.3 °C was obtained, indicating that BIC and DTX formed a homogeneous co-amorphous single phase, i.e., a glass solution [26,27]. 

### 2.2. In Vitro Dissolution Testing

**Figure 2:**
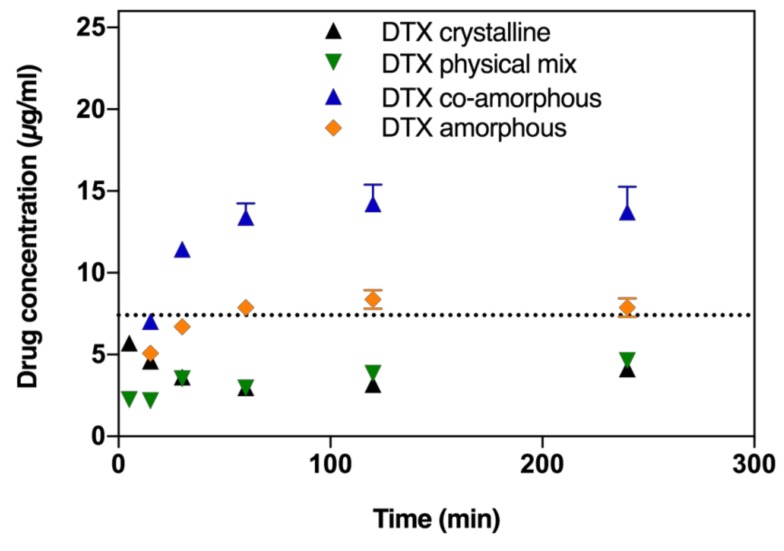
DTX dissolution profiles in fasted state simulated intestinal fluid (FaSSIF) biorelevant medium of crystalline DTX, amorphous ball-milled DTX, physical mixture of crystalline DTX and BIC (at a 1:1 molar ratio), and co-amorphous formulations of DTX and BIC (at a 1:1 molar ratio). Lines represent saturation solubilities. Values are presented as mean ± standard deviation (SD), *n* ≥ 3.

In order to determine whether the amorphous formulations show a supersaturation behaviour, initially the saturation solubilities of the crystalline drugs in fasted state simulated intestinal fluid (FaSSIF) were determined as 3.05 ± 0.9 and 7.5 ± 1.2 µg/mL for BIC and DTX, respectively (determined after 72 h with excess drug powder). Next, the biorelevant dissolution profiles of DTX in the co-amorphous formulation and amorphous DTX were compared to the dissolution of its crystalline counterpart and a crystalline physical mixture with BIC. From Figure 2, it can be seen that the dissolution rates of DTX from the co-amorphous formulation as well as amorphous DTX are both increased compared to the dissolution of DTX from its crystalline powder or in a physical mixture with crystalline BIC. Amorphous DTX appears to dissolve up to the saturation solubility of DTX in FaSSIF (dashed line) but does not supersaturate. On the other hand, from the co-amorphous formulation, DTX is supersaturating already after 30 min and reaches a 1.9-fold degree of supersaturation compared to the saturation solubility after 120 min.

**Figure 3:**
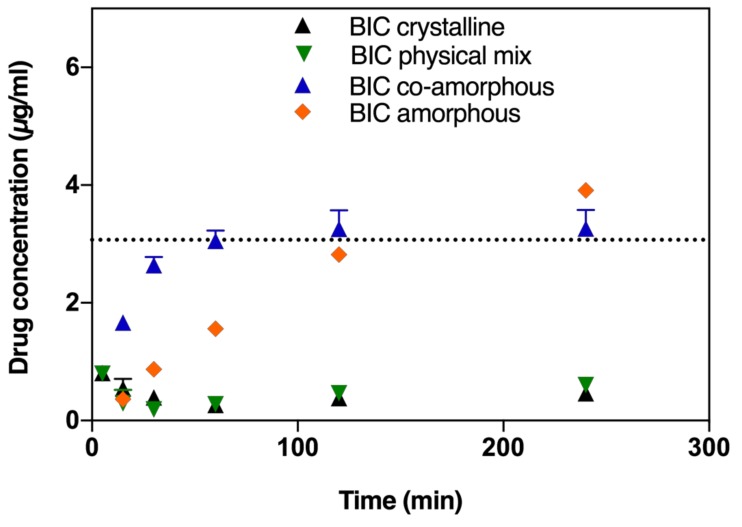
BIC dissolution profiles in FaSSIF biorelevant medium of crystalline BIC, amorphous ball-milled BIC, physical mixture of crystalline DTX and BIC (at a 1:1 molar ratio), and co-amorphous formulations of DTX and BIC (at a 1:1 molar ratio). Lines represent saturation solubilities. Values are presented as mean ± SD, *n* ≥ 3.

Similarly, the BIC dissolution from the co-amorphous formulation was faster compared to pure amorphous BIC, pure crystalline BIC or from a physical mixture of crystalline DTX and BIC (Figure 3). However, BIC from the co-amorphous formulation does not supersaturate but only reaches the saturation solubility of BIC in FaSSIF after 60 min. The dissolution of amorphous BIC is comparatively slower and the saturation solubility is only reached after 120 min.

### 2.3. Physical Stability

**Figure 4:**
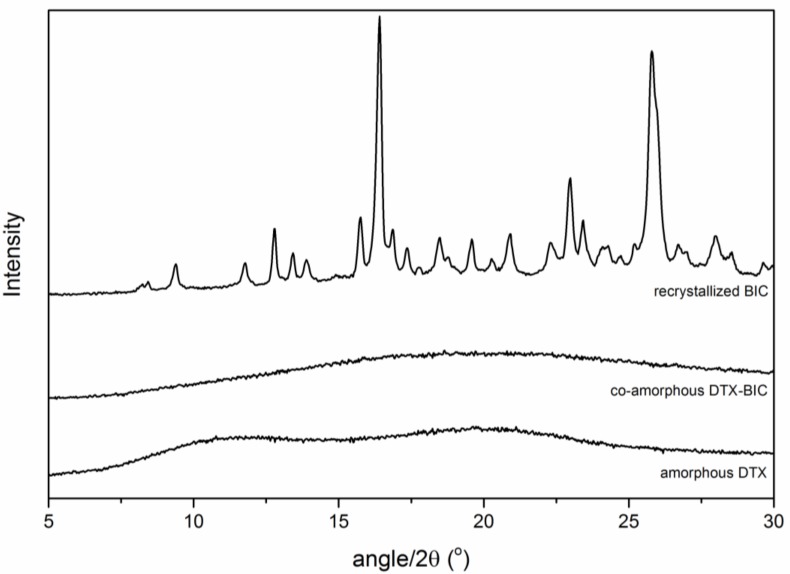
XRPD diffractograms of the ball milled samples DTX, BIC-DTX and BIC after storage for 1.5 years at 4 °C.

Amorphous BIC, amorphous DTX and the co-amorphous BIC-DTX were stored under dry conditions at 4 °C and Figure 4 shows the obtained XRPD patterns after 1.5 years of storage. Both, amorphous DTX and the co-amorphous BIC-DTX showed a characteristic halo pattern, indicating that these two samples remained amorphous. On the other hand, the BIC sample showed distinct diffraction peaks indicating that it had recrystallized during the storage time.

### 2.4. In Vivo Performance

**Figure 5:**
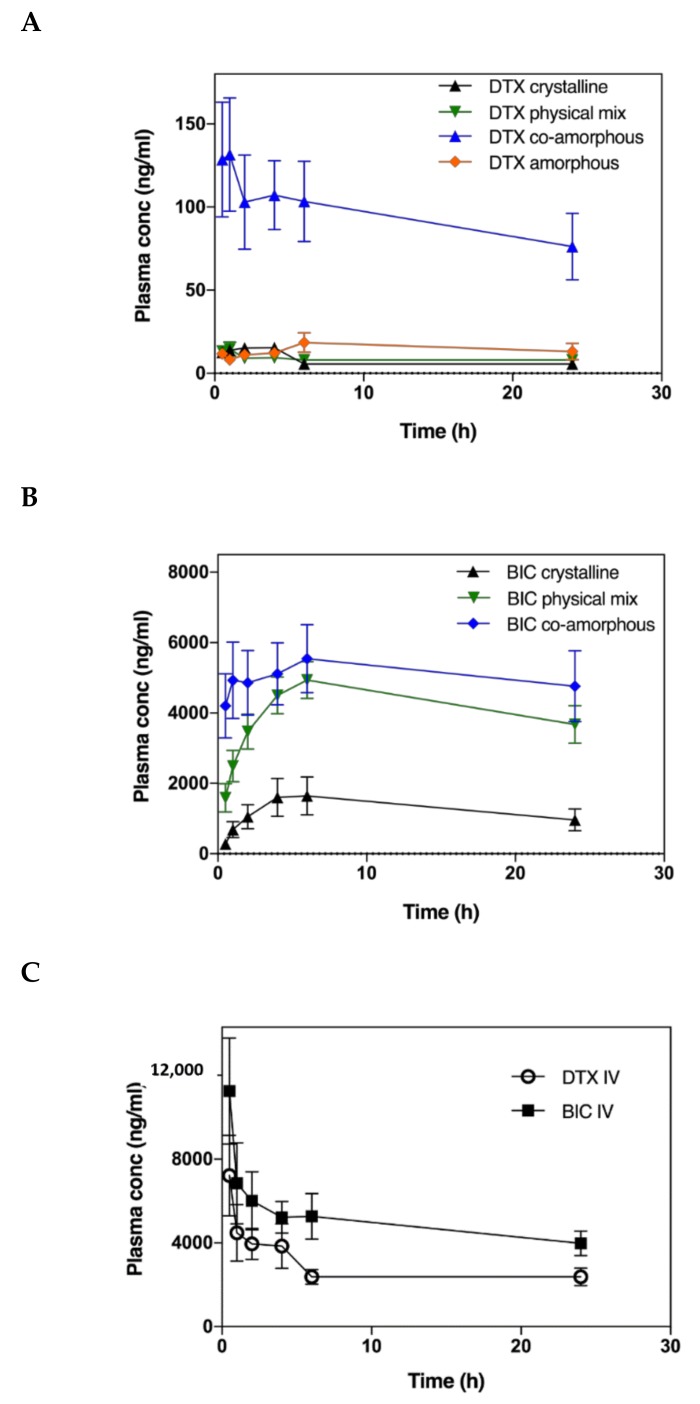
Plasma concentration-time profiles of DTX (**A**) and BIC (**B**) samples in fasted rats after oral administration of drug suspensions and plasma concentration-time profiles of intravenously administered DTX and BIC (**C**). Values are presented as mean ± SEM, *n* ≥ 6.

The pharmacokinetics study investigated the potential improvement in oral bioavailability (% F_a_) of the co-amorphous DTX-BIC mixtures and whether the co-administration of the two drugs, either as a physical mixture or a molecular co-amorphous dispersion, has an impact on the pharmacokinetics of DTX and BIC. The plasma curves for DTX and BIC show that the co-amorphous samples resulted in a quicker onset compared with the other samples (Figure 5). Especially for DTX, the co-amorphous samples showed substantially higher plasma concentrations over the entire study. This could be explained by the faster dissolution observed for the co-amorphous DTX and BIC samples resulting in a higher drug absorption in the early phase of the study. The DTX concentration peaked relatively early around 1 h for the co-amorphous and crystalline samples and stayed relatively stable during the course of the study for all samples, indicating a continuous DTX absorption compared with the intravenous bolus administration (Figure 5C). The BIC concentration increased over a longer time before decreasing again with a T_max_ of 6 h for all samples compared with the earlier T_max_ values observed for DTX (Table 1).

**Figure 6:**
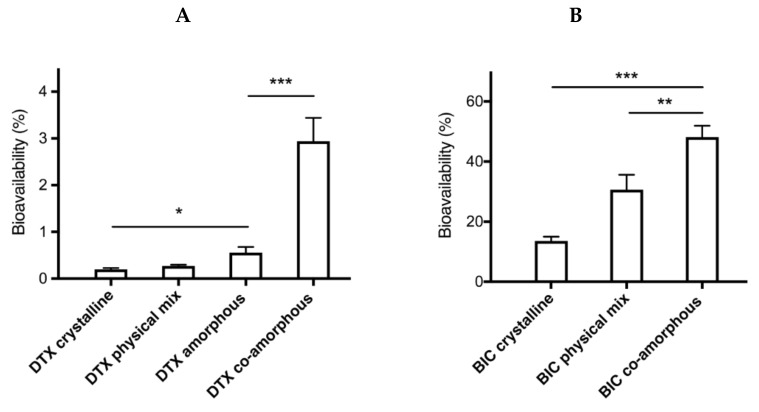
Bioavailability of DTX (**A**) and BIC (**B**) samples in fasted rats after oral administration of drug suspensions. Values are presented as mean ± SEM, *n* ≥ 6. Statistical significance, * *p* < 0.05, ** *p* < 0.01, *** *p* < 0.001.

The C_max_ values observed for DTX were 9 times higher for the co-amorphous samples at 132 ng/mL compared to the crystalline samples at 15 ng/mL. Similarly, the C_max_ values observed for BIC were more than 3 times higher for the co-amorphous samples at 5542 ng/mL compared to the crystalline counterpart at 1664 ng/mL. Moreover, of these orally administered drug formulations, the co-amorphous DTX resulted in the highest bioavailability followed by amorphous DTX, crystalline physical mixture of DTX-BIC, and crystalline DTX (Figure 6, Table 1) (F = 27.85, *p* < 0.00001). The bioavailability observed for co-amorphous DTX was 15-fold higher than for crystalline DTX (and 11-fold higher than the crystalline physical mixture with DTX. Furthermore, the bioavailability of DTX from the co-amorphous formulation was 4.2-fold higher compared to amorphous DTX alone. Interestingly, the crystalline physical mixture of DTX-BIC did not show a significant difference in bioavailability compared with the crystalline DTX. This suggests that the differences in DTX plasma profile observed between the physical mixture and the co-amorphous samples could be associated with the higher dissolution rate and solubility of the co-amorphous samples. It also suggests that the differences in DTX plasma profile observed between the amorphous DTX and the co-amorphous samples could be associated with a permeation enhancing effect of BIC on DTX. The trend is somewhat in agreement with the dissolution data indicating that the co-amorphous formulation with its faster dissolution led to more dissolved drug molecules available for absorption over the intestinal membrane, which is reflected in the higher bioavailability observed.

Further, the results indicate that the combination of DTX and BIC in a co-amorphous formulation had a positive influence on the bioavailability of DTX and BIC. The presence of BIC seems to have an effect on the absorption of dissolved DTX resulting in a higher DTX bioavailability. This is believed to be due to the effect that BIC has on the efflux channels acting as an efflux inhibitor, keeping the efflux channels occupied, and thereby reducing the efflux of DTX which is a substrate to the efflux channels. Hence, compared to pure amorphous DTX, a much higher increase in bioavailability of DTX from the co-amorphous formulation was observed as one would have expected from the in vitro dissolution experiments (approx. 1.9-fold higher solubility).

When comparing with related studies in literature concerning oral drug delivery strategies for DTX, the bioavailability values obtained in this study were lower [19,20], although the C_max_ values were similar. This could be explained by a higher plasma exposure obtained for intravenously administered samples in the present study, resulting in lower bioavailability. Valicherla et al. reported a C_max_ of 125 ng/mL and a 3.2-fold relative bioavailability increase in rats, whereas Zhang et al. reported a C_max_ of 292 ng/mL and a 4.6-fold increase in mice. In our study we found that the co-amorphous formulation of DTX and BIC resulted in a 15-fold increase in bioavailability compared with the crystalline control and a C_max_ of 132 ng/mL compared with 15 ng/mL for the crystalline control.

A similar trend is observed for the bioavailability of BIC, where the highest bioavailability was observed for co-amorphous BIC followed by the crystalline physical mixture of DTX-BIC and crystalline BIC (F = 17.22, *p* < 0.00005). The differences in bioavailability for BIC were more modest compared with DTX as only a 3-fold increase was observed for co-amorphous BIC compared with crystalline BIC and a 2-fold increase was observed for BIC from the crystalline physical mixtures of DTX-BIC compared with crystalline BIC. The increase in the bioavailability of BIC also correlated somewhat with the increased dissolution observed of BIC from the co-amorphous formulation, whereas the crystalline physical mixture with BIC resulted in a high bioavailability despite its poor dissolution rate. This also indicates that, in addition to the dissolution behaviour, the efflux substrate-inhibitor synergy between DTX and BIC had a positive influence on the intestinal absorption of BIC. In addition to its mechanism as an efflux inhibitor, BIC also acts as an inhibitor of CYP3A4 and CYP2C19 enzymes, which could further explain its positive influence on the oral bioavailability of DTX [22]. However, additional experiments are necessary to investigate the precise contribution of the efflux inhibition or CYP inhibition on the overall absorption enhancement.

## 3. Materials and Methods

### 3.1. Materials

Docetaxel (DTX, Mw = 807.9 g/mol, 99.4% purity) was obtained from Xingcheng Chempharm (Zhejiang, China) and bicalutamide (BIC, Mw = 430.3 g/mol, 99.7% purity) was obtained from Taizhou Creating Chemical (Taizhou, China). Bovine bile extract, Tween 80, dimethyl sulfoxide and Tris maleate salt were purchased from Sigma-Aldrich (St. Louis, MO, USA), phosphatidylcholine (99%) was purchased from Lipoid (Ludwigshafen, Germany), and sodium chloride (99.5%) was purchased from Fluka Chemie AG (Buchs, Switzerland). Phosphate buffered saline (PBS) and fasted state simulated intestinal fluid (FaSSIF) were purchased from Corning (Midland, MI, USA) and Biorelevant (London, UK), respectively. Ultrapure water (SG Water Purification System, Barsbuttel, Germany) was used for all experiments. All other chemicals and solvents were of analytical grade and were used without further purification.

### 3.2. Preparation of the Amorphous Materials

Vibrational ball milling (BM) was applied to obtain the amorphous pure drugs and the co-amorphous DTX-BIC mixture. Briefly, the samples were prepared by adding a total mass of 500 mg of the appropriate amount of DTX, BIC or DTX and BIC at a 1:1 molar ratio in 25 mL milling jars using two stainless steel balls with a diameter of 12 mm, and subsequent milling for a duration of 120 min, at 30 Hz in a cold room at +6 °C using an oscillatory ball mill (MixerMill MM400, Retsch GmbH & Co., Haan, Germany). All BM powders were prepared in triplicate and used for in vitro and in vivo testing within 24 h after preparation. 

### 3.3. X-Ray Powder Diffraction (XRPD)

XRPD was performed using an X’Pert PANalytical PRO X-ray diffractometer (PANalytical, Almelo, The Netherlands) using Cu Kα radiation (λ = 1.54 Å), and an acceleration voltage and current of 45 kV and 40 mA, respectively. The powder samples were scanned in reflectance mode from 5° to 35° 2ϴ with a scan speed of 0.067° 2ϴ and a step size of 0.026° 2ϴ. Data were collected and analyzed using the software X’Pert Data Collector (PANalytical, Almelo, The Netherlands).

### 3.4. Differential Scanning Calorimetry (DSC)

DSC measurements were performed using a Discovery DSC (TA Instruments, New Castle, DE, USA) in the modulated temperature mode. Samples of approximately 5 mg were weighed into Tzero aluminum pans and sealed with a Tzero lid. Measurements were performed from −10 to 190 °C with a heating rate of 2 °C/min, a modulation amplitude of 0.2120 °C and a period of 40 s. A constant nitrogen flow rate of 50 mL/min was used during the measurements. The glass transition temperatures (*T_g_*, midpoint) were determined as the mean of three independent measurements from the reversing heat flow signal. Data analysis was performed using the Trios software (TA Instruments-waters LLC, New Castle, DE, USA). 

### 3.5. Saturation Solubility Studies

DTX and BIC saturation solubility studies were conducted in FaSSIF. The biorelevant FaSSIF medium was prepared by adding 218 mg bovine bile extract, 58 mg phosphatidylcholine, 232 mg Tris maleate salt and 395 mg sodium chloride to 100 mL of ultra-purified water and stirring overnight at 37 °C. The medium pH was adjusted to 6.5 with 1 M NaOH before use. Approximately 100 mg of the drugs were mixed with 1.5 mL of the media in plastic tubes, and the suspensions were rotated at 20 rpm for 72 h. The solution was separated from undissolved drug by centrifugation, and aliquots from the saturated solution were filtered through 0.45 µm polyvinylidene difluoride (PVDF) filters. The solubility may potentially have been influenced by exposure to temperature since both, centrifugation and filtration, were conducted at room temperature. 

The concentrations of DTX and BIC were determined using a reversed-phase high-performance liquid chromatography (HPLC) system (Dionex Corporation, CA, USA) equipped with an ASI-100 autosampler, Dionex Ultimate 300 UV-Visible detector and pump. Chromatographic separation was achieved using a XB-C18 Phenomenex Kinetex column of 2.6 μm (2.1 × 50 mm) (Torrance, CA, USA) at 30 °C. The mobile-phase consisted of a mixture of A) 95% water 5% acetonitrile (ACN) and 0.1% trifluoroacetic acid (TFA) and B) 95% ACN 5% water 0.1% TFA, and was used at a flow rate of 0.5 mL/min in isocratic mode (60% A 40% B). Peaks were analysed at 270 nm for BIC and 230 nm for DTX. Linear calibration curves in the range of 0.25–5 μg/mL for both BIC and DTX were obtained in acetonitrile. Elution times were 4.8 min for BIC and 6.4 min for DTX.

### 3.6. Dissolution Studies

For the dissolution studies, powder formulations were initially suspended in PBS in order to avoid powder agglomerates. Immediately after suspension, 100 µL aliquots were added to each well of a 12-well plate containing 2.9 mL dissolution medium/well. The concentrations of the drugs in the dissolution medium were 3 times higher than their respective saturation solubilities. The plates were closed with a lid to limit evaporation and incubated at 37 °C for 4 h in an orbital shaker with a rotation speed of 150 rpm. At predetermined time points (15, 30, 60, 120 and 240 min), 50 μL aliquots were withdrawn and immediately replaced with dissolution medium. The obtained samples were centrifuged at 10,000 *g* for 10 min and the supernatant was then analysed by HPLC (see above). All dissolution experiments were conducted in triplicate.

### 3.7. Stability Studies

The co-amorphous binary DTX-BIC mixture as well as the pure individual amorphous drugs were stored in a desiccator under dry conditions (silica gel) at 4 °C and analysed by XRPD after 1.5 years of storage. 

### 3.8. In Vivo Pharmacokinetics Studies in Rats

Healthy female and male Sprague Dawley rats of 7 weeks age weighing 140–240 g (Charles River, Denmark), were used for the experiments. The rats were housed with free access to water and food in groups of two rats per cage under controlled environmental conditions (constant temperature and humidity with a 12 h day-night cycle). All animal experimental work was carried out under the protocol approved by the Danish Animal Experiments Inspectorate (approval no. 2014-15-0201-00031), and all the procedures used were refined to provide maximal comfort and minimal stress to the animals.

All animals were fasted for approximately 12 h prior to administration of the drug formulations and food was provided to rats approximately 6 h after drug administration, although free access to water was allowed during fasting. The nine groups studied, each consisting of 6–8 rats, were assigned randomly and included two groups receiving an intravenous bolus injection of either DTX or BIC corresponding to 1 mg/rat (4–7 mg/kg) dissolved in saline: DMSO (50:50 *v*/*v*) with 1% Tween 80, administered in the tail vein. The remaining seven groups were orally administered with DTX or BIC or a combination of these at a fixed dose of 30 mg/kg for DTX and 16 mg/kg for BIC. For DTX, four groups of animals were studied, one group administered with crystalline DTX (crystalline), one group administered with a physical mixture of crystalline DTX and BIC (physical mix), one group administered with amorphous DTX (amorphous) and one group administered with the co-amorphous mixture of DTX and BIC (co-amorphous). For BIC three groups of animals were studied, one group administered with crystalline BIC (crystalline), one groups administered with a physical mixture of crystalline DTX and BIC (physical mix) and one group administered with the co-amorphous mixture of DTX and BIC (co-amorphous). Drug powders were suspended in 0.5 mL PBS and the suspension was immediately administered to the rats using a flexible cannula. Blood samples (150–200 µL) were collected in EDTA coated tubes from puncturing of the tail vein at 0.5, 1, 2, 4, 6 and 24 h after drug administration. The rats were euthanized by cervical dislocation after collection of the final blood sample. Plasma samples were obtained by centrifugation of blood samples at 3600 g (10 min, 4 °C) and transferred into microtubes. The plasma samples were stored at −80 °C until used for further analysis.

### 3.9. Quantification of Plasma Samples

The bioanalysis of DTX and BIC in rat plasma was carried out using HPLC coupled with mass spectrometric (MS) detection. Prior to analysis liquid-liquid extraction was performed on the plasma samples (50 μL) by adding 100 μL of ethyl acetate and subsequently vortexing the vial for protein precipitation. The vials were centrifuged and 100 μL supernatant was carefully transferred to HPLC vials with inserts and evaporated using compressed air. The dried content of the vial was reconstituted using 150 μL ACN:H2O (1:1 *v*/*v*) and measured by LC-MS/MS. 

LC-MS/MS analysis was performed using a 1290 Agilent Infinity Series system (Agilent Technologies, Palo Alto, CA, USA) coupled to an AB Sciex 4500 QTrap MS (Applied Biosystems, Foster City, CA, USA). A HPLC-MS method was developed for each compound. In both cases chromatographic separation was performed using a Phenomenex Kinetex (Torrance, CA, USA) C18 (50 × 2.1 mm, 2.6 μm) column with a Phenomenex C18 SecurityGuard™ Ultra (2.1 mm, 2 µm) guard column and the mobile phases consisted of (A) MilliQ water with 0.1% formic acid and (B) ACN with 0.1% formic acid. The column and autosampler tray were set at 40 °C and 6 °C, respectively, and the flow rate was set at 0.25 mL/min. The injection volume was set at 1 μL and 5 μL for BIC and DTX, respectively. For BIC, the elution gradient started at 20% B at time 0 and increased to 70% B at time 3.0 min. Hereafter B was held at 99% until 4.3 min before re-equilibrating the column until 5.5 min. The retention time for BIC was 3.12 min. For DTX, the elution gradient started at 20% B at time 0 and increased to 75% B at time 3.5 min. Hereafter, B was held at 99% until 4.3 min before re-equilibrating the column until 6 min. The retention time for BIC was 3.51 min.

In both methods the MS source parameters were: 450 °C turbo gas temperature, 30 mL/min curtain gas (N_2_), collision gas was set to High, 40 mL/min for gas 1(N_2_) and 30 mL/min for gas 2(N_2_). Ionspray voltage was set at −4500 V and 5500 V for BIC and DTX, respectively. The MS compound dependent parameters for BIC quantifier 428.8 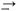
254.6 m/z and qualifier 428.8 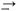
184.5 m/z ions were as follows: collision energy of −23 V and −42 V, collision cell exit potential of −10 V and −6 V, respectively, and with a declustering potential of −80 V and an entrance potential of −10V for both ions. The MS compound-dependent parameters for DTX quantifier 830.5 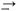
549.1 m/z and qualifier 830.5 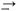
248.0 m/z ions were as follows: collision energy of 36 V and 41 V, collision cell exit potential of 22 V and 13 V, respectively, and with a declustering potential of 140 V and an entrance potential of 10V for both ions. 

A calibration curve was constructed with 7 calibration points in triplicates using DTX and BIC. Calibration standards were prepared by spiking blank rat plasma with different concentrations of DTX and BIC over a range of 1–1000 ng/mL.

### 3.10. Pharmacokinetic Analysis

The area under the curve (AUC) for the plasma concentration profile was determined and was used to calculate the absolute oral bioavailability (F_a_). The oral bioavailability (in %) was calculated as, F_a_ = AUC_oral_/AUC_IV_ × 100. The maximum plasma concentration C_max_ and the time T_max_, at which C_max_ was detected, were determined from the individual plasma concentration curves.

### 3.11. Statistical Analysis

All in vitro experiments/measurements were carried out in triplicate and values are presented as the mean ± standard deviation (SD) unless otherwise noted. All in vivo data are presented as mean ± standard error of the mean (SEM) unless otherwise stated, and statistical significance between samples was evaluated using Student’s t-test and one-way ANOVA with Tukey’s test. 

## 4. Conclusions

This study highlighted the potential of preparing co-amorphous formulations of anticancer drugs to increase their biological activity in combination treatments. It was possible to prepare a stable co-amorphous system of DTX and BIC at a molar ratio of 1:1, that showed both improved in vitro and in vivo performance for both drugs. The applied co-amorphous formulation approach made it possible to prepare a fast dissolving and supersaturating drug delivery system for the poorly soluble drug DTX. Furthermore, by co-formulating the efflux pump substrate DTX with an efflux pump inhibitor BIC into a co-amorphous mixture, the bioavailability of DTX could be increased significantly compared to the pure crystalline and amorphous drug as well as a physical mixture of DTX and BIC. Such an approach of combining two complimentary drugs for combination therapy has the further advantage that the two drugs are stabilizing each other in an amorphous form, hence, acting as active and amorphous stabilizer at the same time, omitting the need for any stabilizing excipient. Overall, the results suggest that such a formulation approach has the potential of increasing DTX bioavailability after oral administration, providing better in vivo performance of DTX in cancer treatments. 

## Figures and Tables

**Table 1:** Summary of the pharmacokinetic parameters of DTX and BIC samples in fasted rats after oral administration of drug suspensions. Values are presented as mean ± standard error of the mean (SEM), *n* ≥ 6.

	DTX				BIC			
	AUC (ng/mL × 24h)	Fa (%)	C_max_ (ng/mL)	T_max_ (h)	AUC (µg/mL × 24h)	Fa (%)	C_max_ (ng/mL)	T_max_ (h)
Crystalline	185 ± 29	0.20 ± 0.03	15	4	43.06 ± 4.79	14 ± 1	1664	6
Physical mix	253 ± 29	0.27 ± 0.02	15	1	97.21 ± 15.80	31 ± 5	4937	6
Co-amorphous	2188 ± 264	2.94 ± 0.50	132	1	138.72 ± 11.12	48 ± 4	5542	6
Amorphous	518 ± 113	0.56 ± 0.12	19	6	^*^ N/A	N/A	N/A	N/A
Intravenous	93,196 ± 12,626	N/A	7212	0.5	317,208 ± 20,888	N/A	11,253	0.5

^*^ N/A: not applicable

## Supplementary Materials

The following are available online. Figure S1: Thermograms of amorphous DTX, amorphous BIC and co-amorphous DTX-BIC after ball milling.
