# Supplementary material for: Efflux Inhibitor Bicalutamide Increases Oral Bioavailability of the Poorly Soluble Efflux Substrate Docetaxel in Co-Amorphous Anti-Cancer Combination Therapy
Source: Molecules. 2019 Jan 11;24(2):266. doi: 10.3390/molecules24020266 (PMC6369428; doi:10.3390/molecules24020266)
Supplement: Supplementary file 1 [file molecules-24-00266-s001.pdf]

Supplementary data

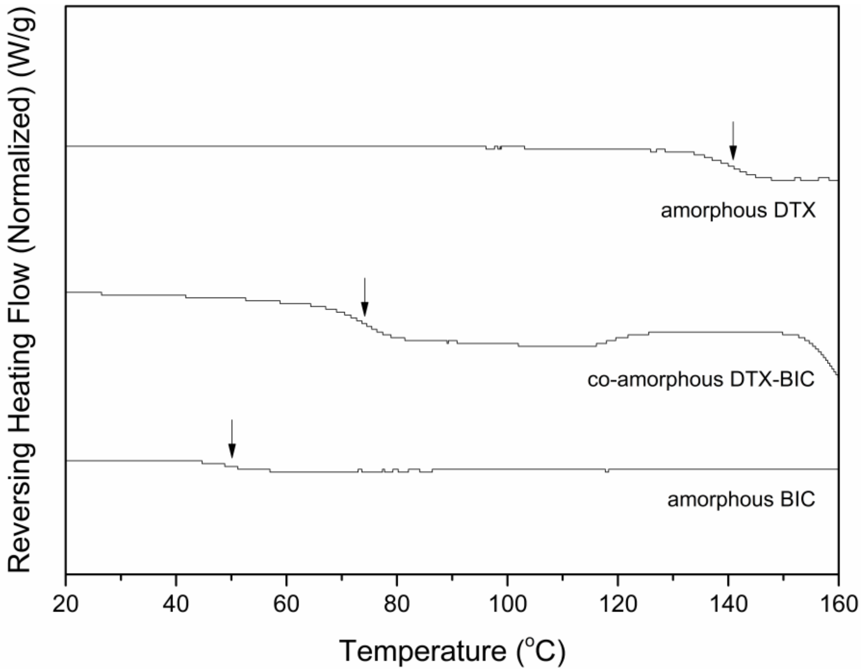

Figure S1: Thermograms of amorphous DTX, amorphous BIC and co-amorphous DTX-BIC after ball milling.
